# The Tip of The Iceberg: Non-Calcified Coronary Plague and Epicardial Adipose Tissue

**DOI:** 10.5935/abc.20170046

**Published:** 2017-04

**Authors:** Levent Cerit

**Affiliations:** Near East University-Nicosia-Cyprus

**Keywords:** Adipose Tissue / pathology, Coronary Artery Disease, Calcium Signaling, Biomarkers / analysis, Radionuclide Imaging, Tomography, Emission Computed

**To the Editor,**

I have read with great interest the article entitled "Relationship between Calcium Score and Myocardial Scintigraphy in the Diagnosis of Coronary Disease" by Siqueira et al.,^1^ recently published in *Arquivos Brasileiros de Cardiologia* 2016; 107:367-74. The investigators reported the possibility of removing extensive coronary artery disease (CAD) by means of a zero calcium score, or by indicating the presence of an extensive disease when it is severely increased, which justifies the use of this method in the initial or joint evaluation in asymptomatic patients with suspected CAD and in cardiovascular risk stratification. The evaluation of symptomatic low-risk patients, despite suggestive evidence, should be re-evaluated in upcoming guidelines.^1^

Epicardial adipose tissue (EAT) is anatomically contiguous with the myocardium and several studies have shown it to be a potential contributing factor for coronary atherosclerosis.^2^ EAT is a type of visceral adipose tissue with paracrine and endocrine effects.^3^ EAT serves as an energy source for the myocardium and it is known to secrete proatherogenic cytokines.^3^ Increased EAT is not only associated with a higher prevalence of CAD but it is also a prognostic parameter for future cardiovascular events, and, eventually, cardiovascular mortality.^4^ Hwang et al.^5^ have reported that a high epicardial fat volume index determined by computed tomography was an independent risk factor for the future development of non-calcified coronary plaque even after adjustment for traditional cardiovascular risk factors.

In the light of these findings, assessment of EAT by computed tomography might be beneficial as a part of further evaluation for future cardiovascular events.

## References

[1] Siqueira FP, Mesquita CT, Santos AA, Nacif MS (2016). Relationship between calcium score and myocardial scintigraphy in
the diagnosis of coronary disease. Arq Bras Cardiol.

[2] Lee HY, Després JP, Koh KK (2013). Perivascular adipose tissue in the pathogenesis of cardiovascular
disease. Atherosclerosis.

[3] Mazurek T, Zhang L, Zalewski A, Mannion JD, Diehl JT, Arafat H (2003). Human epicardial adipose tissue is a source of inflammatory
mediators. Circulation.

[4] Mahabadi AA, Berg MH, Lehmann N, Kälsch H, Bauer M, Kara K (2013). Association of epicardial fat with cardiovascular risk factors
and incident myocardial infarction in the general population: the Heinz
Nixdorf Recall Study. J Am Coll Cardiol.

[5] Hwang IC, Park HE, Choi SY (2016). Epicardial adipose tissue contributes to the development of
non-calcified coronary plaque: a 5-year computed tomography follow-up
study. J Atheroscler Thromb.

