# Supplementary material for: The Tip of The Iceberg: Non-Calcified Coronary Plague and Epicardial Adipose Tissue
Source: Arq Bras Cardiol. 2017 Apr;108(4):383–5. doi: 10.5935/abc.20170046 (PMC5421481; doi:10.5935/abc.20170046)
Supplement: Related texts

# Reply

**Authors:** Fabio Paiva Rossini Siqueira, Claudio Tinoco Mesquita, Alair Augusto Sarmet M. Damas dos Santos, Marcelo Souto Nacif

We would like to thank you for your interest and comments related to our recent article.^1^ We believe the great scientific basis referent to coronary calcium quantification clearly demonstrates the importance of this method in the stratification of asymptomatic patients with low to intermediate cardiovascular risk.^2-7^

Recent publications related to calcium score (CS) continue to point to flaws in clinical score classifications and reinforce their ability to distinguish the different cardiovascular events risk groups. The role of clinical score reclassification, when CS is implemented, is a warning to its clinical applicability.^2-7^ Population studies with long periods of follow-up^2-7^ have demonstrated that the use of CS is one of the best tools to determine cardiovascular risk, even when compared to other markers.^7^

The potential use of CS is not limited to cardiovascular risk evaluation, for it has proven to be a useful tool in the primary prevention and adequate treatment of sub-clinical forms of coronary artery disease.^8-15^ The presence of a zero CS is indicative of a very low risk, often exempting the patient from early preventive treatment with statins.^8-11^ On the other hand, in the presence of a CS that is not zero, and especially > 100 Agatston, therapeutic introduction may be recommended, even in patients who do not fit the indication of current guidelines for the use of statins and other medications, such as anti-hypertensives.^10-15^

Important changes in the guidelines can already be seen, putting CS in the recommendation class I for some of its indications.^16^ However, there still are some discrepant recommendations,^17-20^ such as in orientations about the beginning of treatment for cardiovascular risk reduction, that still do not include coronary calcification data, even with robust data that support this positioning.^21-22^

Therefore, we believe that in the next few years, this method will take on a growing importance in clinical guidelines, aiding in a more adequate follow-up of low to intermediate risk patients.

Yours truly,

**Fabio Paiva Rossini Siqueira,**

**Claudio Tinoco Mesquita,**

**Alair Augusto Sarmet M. Damas dos Santos,**

**Marcelo Souto Nacif**

## References

[1] Siqueira FP, Mesquita CT, Santos AA, Nacif MS (2016). Relationship between calcium score and myocardial
scintigraphy in the diagnosis of coronary disease. Arq Bras Cardiol.

[2] Valenti V, Ó Hartaigh B, Heo R, Cho I, Schulman-Marcus J, Gransar H (2015). A 15-year warranty period for asymptomatic individuals
without coronary artery calcium: a prospective follow-up of 9,715
individuals. JACC Cardiovasc Imaging.

[3] Shaw LJ, Giambrone AE, Blaha MJ, Knapper JT, Berman DS, Bellam N (2015). Long-term prognosis after coronary artery calcification
testing in asymptomatic patients: a cohort study. Ann Intern Med.

[4] McClelland RL, Jorgensen NW, Budoff M, Blaha MJ, Post WS, Kronmal RA (2015). 10-year coronary heart disease risk prediction using coronary
artery calcium and traditional risk factors: derivation in the MESA
(Multi-Ethnic Study of Atherosclerosis) with validation in the HNR
(Heinz Nixdorf Recall) Study and the DHS (Dallas Heart
Study). J Am Coll Cardiol.

[5] Blaha MJ, Cainzos-Achirica M, Greenland P, McEvoy JW, Blankstein R, Budoff MJ (2016). Role of coronary artery calcium score of zero and other
negative risk markers for cardiovascular disease: the Multi-Ethnic Study
of Atherosclerosis (MESA). Circulation.

[6] Diederichsen AC, Mahabadi AA, Gerke O, Lehmann N, Sand NP, Moebus S (2015). Increased discordance between HeartScore and coronary artery
calcification score after introduction of the new ESC prevention
guidelines. Atherosclerosis.

[7] Joshi PH, Patel B, Blaha MJ, Berry JD, Blankstein R, Budoff MJ (2016). Coronary artery calcium predicts cardiovascular events in
participants with a low lifetime risk of cardiovascular disease: the
Multi-Ethnic Study of Atherosclerosis (MESA). Atherosclerosis.

[8] Pursnani A, Massaro JM, D'Agostino RB, O'Donnell CJ, Hoffmann U (2015). Guideline-based statin eligibility, coronary artery
calcification, and cardiovascular events. JAMA.

[9] Nasir K, Bittencourt MS, Blaha MJ, Blankstein R, Agatson AS, Rivera JJ (2015). Implications of coronary artery calcium testing among statin
candidates according to American College of Cardiology/American Heart
Association Cholesterol Management Guidelines: MESA (Multi-Ethnic Study
of Atherosclerosis). J Am Coll Cardiol.

[10] Isma'eel H, Min D, Al-Shaar L, Hachamovitch R, Halliburton S, Gentry J (2016). Assessing Level of Agreement for Atherosclerotic
Cardiovascular Disease Risk Categorization Between Coronary Artery
Calcium Score and the American College of Cardiology/American Heart
Association Cardiovascular Prevention Guidelines and the Potential
Impa. Am J Cardiol.

[11] Waheed S, Pollack S, Roth M, Reichek N, Guerci A, Cao JJ (2016). Collective impact of conventional cardiovascular risk factors
and coronary calcium score on clinical outcomes with or without statin
therapy: The St Francis Heart Study. Atherosclerosis.

[12] Martin SS, Blaha MJ, Blankstein R, Agatston A, Rivera JJ, Virani SS (2014). Dyslipidemia, coronary artery calcium, and incident
atherosclerotic cardiovascular disease: implications for statin therapy
from the multi-ethnic study of atherosclerosis. Circulation.

[13] McEvoy JW, Martin SS, Dardari ZA, Miedema MD, Sandfort V, Yeboah J (2017). Coronary artery calcium to guide a personalized risk-based
approach to initiation and intensification of antihypertensive
therapy. Circulation.

[14] Miedema MD, Duprez DA, Misialek JR, Blaha MJ, Nasir K, Silverman MG (2014). Use of coronary artery calcium testing to guide aspirin
utilization for primary prevention: estimates from the multi-ethnic
study of atherosclerosis. Circ Cardiovasc Qual Outcomes.

[15] Darabian S, Luo Y, Homat A, Khosraviani K, Wong N, Zeb I (2015). CAC score as a possible criterion for administration of
angiotensin converting enzyme inhibitors and/or angiotensin receptor
blockers the MultiEthnic Study of Atherosclerosis. Coron Artery Dis.

[16] Sara L, Szarf G, Tachibana A, Shiozaki AA, Villa AV, de Oliveira AC, Sociedade Brasileira de Cardiologia, Colégio Brasileiro de Radiologia (2014). II Guidelines on Cardiovascular Magnetic Resonance and
Computed Tomography of the Brazilian Society of Cardiology and the
Brazilian College of Radiology. Arq Bras Cardiol.

[17] Piepoli MF, Hoes AW, Agewall S, Albus C, Brotons C, Catapano AL (2016). 2016 European Guidelines on cardiovascular disease prevention
in clinical practice: The Sixth Joint Task Force of the European Society
of Cardiology and Other Societies on Cardiovascular Disease Prevention
in Clinical Practice (constituted by representatives of 10 societies and
by invited experts)Developed with the special contribution of the
European Association for Cardiovascular Prevention & Rehabilitation
(EACPR). Eur Heart J.

[18] Mahabadi AA, Möhlenkamp S, Lehmann N, Kälsch H, Dykun I, Pundt N, Heinz Nixdorf Recall Study Investigators (2017). CAC score improves coronary and CV risk assessment above
statin indication by ESC and AHA/ACC Primary Prevention
Guidelines. JACC Cardiovasc Imaging.

[19] Goff DC Jr, Lloyd-Jones DM, Bennett G, Coady S, D'Agostino RB Sr, Gibbons R, American College of Cardiology/American Heart Association Task
Force on Practice Guidelines (2014). 2013 ACC/AHA guideline on the assessment of cardiovascular
risk: a report of the American College of Cardiology/American Heart
Association Task Force on Practice Guidelines. J Am Coll Cardiol.

[20] Wolk MJ, Bailey SR, Doherty JU, Douglas PS, Hendel RC, Kramer CM, American College of Cardiology Foundation Appropriate Use
Criteria Task Force (2014). ACCF/AHA/ASE/ASNC/HFSA/HRS/SCAI/SCCT/SCMR/STS 2013 multi-
modality appropriate use criteria for the detection and risk assessment
of stable ischemic heart disease: a report of the American College of
Cardiology Foundation Appropriate Use Criteria Task Force, American
Heart Association, American Society of Echocardiography, American
Society of Nuclear Cardiology, Heart Failure Society of America, Heart
Rhythm Society, Society for Cardiovascular Angiography and
Interventions, Society of Cardiovascular Computed Tomography, Society
for Cardiovascular Magnetic Resonance, and Society of Thoracic
Surgeons. J Am Coll Cardiol.

[21] Stone NJ, Robinson JG, Lichtenstein AH, Bairey Merz CN, Blum CB, Eckel RH (2014). 2013 ACC/AHA guideline on the treatment of blood cholesterol
to reduce atherosclerotic cardiovascular risk in adults a report of the
American College of Cardiology/American Heart Association Task Force on
Practice Guidelines. J Am Coll Cardiol.

[22] Catapano AL, Graham I, De Backer G, Wiklund O, Chapman MJ, Drexel H (2016). 2016 ESC/EAS Guidelines for the Management of
Dyslipidaemias. Eur Heart J.
